# Increase CO_2_ recycling of *Escherichia coli* containing CBB genes by enhancing solubility of multiple expressed proteins from an operon through temperature reduction

**DOI:** 10.1128/spectrum.02560-23

**Published:** 2023-10-11

**Authors:** Jaeyoung Yu, Woo-Ri Shin, Ji Hun Kim, Soo Youn Lee, Byung-Kwan Cho, Yang-Hoon Kim, Jiho Min

**Affiliations:** 1 Graduate School of Semiconductor and Chemical Engineering, Jeonbuk National University, Jeonju-si, South Korea; 2 Department of Biotechnology and Life Science, Tokyo University of Agriculture and Technology, Tokyo, Japan; 3 School of Biological Sciences, Chungbuk National University, Cheongju, South Korea; 4 Department of Biological Sciences, Korea Advanced Institute of Science and Technology, Daejeon, South Korea; 5 Gwangju Bio/Energy R&D Centre, Korea Institute of Energy Research, Gwangju, South Korea; Istituto Italiano di Tecnologia, Torino, Italy

**Keywords:** biological carbon fixation, Calvin-Benson cycle, inclusion body, integrated autotrophic biorefinery, temperature controls

## Abstract

One of the current promising solutions to address problems of CO_2_ emissions is the development of biological carbon fixation strategies. This strategy leads to higher chemical biosynthetic productivity through improved biological CO_2_ fixation. In a previous study, we introduced the Calvin-Benson Bassham (CBB) genes of the photosynthetic bacterium *Cereibacter sphaeroides* into *Escherichia coli* to develop a strain capable of endogenous CO_2_ recycling by heterologous expression of the CBB genes. However, the heterologous expression of recombinant proteins in *E. coli* is often hampered by inclusion bodies (IB), which are protein aggregates. Various factors contribute to IB formation, including host cell metabolism, protein synthesis, transformation machinery, and target protein properties. In this study, we investigated the influence of environmental conditions, particularly culture temperature, on IB formation and CO_2_ recycling in the CBB strain. As a result, by reducing the culture temperature from 37°C to 30°C, a significant suppression of IB formation was achieved, resulting in a remarkable decrease in CO_2_ release by approximately 5.76 times. In addition, interestingly, an enhancement in the accumulation of pyruvate by approximately 2.3 times was observed at the same time. These results demonstrate the simultaneous improvements in CO_2_ recycling and the synthesis of organic acids achieved through temperature control. Based on the findings of this study, we believe that temperature control would be a promising approach to increasing chemical biosynthetic productivity as well as biological CO_2_ fixation activity in integrated autotrophic biorefinery strategies.

**IMPORTANCE:**

In a previous study, we successfully engineered *Escherichia coli* capable of endogenous CO_2_ recycling through the heterologous expression of the Calvin-Benson Bassham genes. Establishing an efficient gene expression environment for recombinant strains is crucial, on par with the importance of metabolic engineering design. Therefore, the primary objective of this study was to further mitigate greenhouse gas emissions by investigating the effects of culture temperature on the formation of inclusion bodies (IB) and CO_2_ fixation activity in the engineered bacterial strain. The findings demonstrate that lowering the culture temperature effectively suppresses IB formation, enhances CO_2_ recycling, and concurrently increases the accumulation of organic acids. This temperature control approach, without adding or modifying compounds, is both convenient and efficient for enhancing CO_2_ recycling. As such, additional optimization of various environmental parameters holds promise for further enhancing the performance of recombinant strains efficiently.

## INTRODUCTION

Human activities have unconsciously released CO_2_ into the atmosphere using fossil fuels for centuries (1), leading to increased atmospheric CO_2_ levels and subsequent global warming and climate change (2). Nevertheless, due to coal’s cheap and abundant advantages, dependence on fossil fuels and CO_2_ emissions are expected to increase (3). One of the current promising solutions to address the problems of CO_2_ emissions is the development of biological carbon fixation strategies (4). Biological carbon fixation using microorganisms is considered to be environmentally friendly, economically efficient, and sustainable while at the same time producing value-added chemicals (5). Bacteria using the Calvin-Benson Bassham (CBB) cycle for autotrophic CO_2_ fixation can convert CO_2_ in the form of biofuels and biomass (6). However, a single bioprocess with an autotrophic mode has limitations, such as the range of products produced and economic feasibility. Therefore, linking different autotrophic bioprocesses helps to overcome limitations and increase sustainability (7). In recent years, strategies have been developed to establish these integrated autotrophic biorefineries. This strategy increases chemical biosynthetic productivity through improved biological CO_2_ fixation (8). Xia et al. introduced synthetic ribulose-1,5-bisphosphate carboxylase/oxygenase (RuBisCO) and phosphoribulokinase (PRK) genes into *Saccharomyces cerevisiae* to concurrently achieve CO_2_ recycling and lignocellulosic bioethanol synthesis (9). Likewise, Pang et al. introduced RuBisCO and RuBisCO activase genes into *Escherichia coli* to simultaneously accomplish metabolite production (ethanol, acetate, and pyruvate) and CO_2_ recycling (10).

From this point of view, we introduced the CBB genes of the photosynthetic bacterium *Cereibacter sphaeroides* (also known as *Rhodobacter sphaeroides*) into *E. coli* to develop a strain capable of endogenous CO_2_ recycling by heterologous expression of the whole CBB genes (11). However, the heterologous expression of recombinant proteins in *E. coli* is often hampered by inclusion bodies (IB), which are protein aggregates. IB primarily consists of proteins expressed from foreign or mutated genes and occurs due to imbalances in protein folding, aggregation, and degradation processes (12). Various factors contribute to IB formation, including host cell metabolism, protein synthesis, transformation machinery, and target protein properties (12). In this study, we tried to suppress IB formation and increase CO_2_ recycling in the CBB strain by controlling environmental conditions, particularly culture temperature. The findings of this study provide insights into the interplay between culture temperature, IB formation, and CO_2_ recycling in the CBB strain.

## RESULTS AND DISCUSSION

### Suppression of IB formation and reduction of CO_2_ release by temperature control

This study aims to assess the influence of culture temperature on IB formation, providing insights into enhancing CO_2_ recycling in biological carbon fixation strategies. As a control group (MOCK), we used *E. coli* BL21(DE3) containing empty vectors pET-21a and pET-28b. Additionally, the CBB strain refers to *E. coli* BL21(DE3) transformed with pET-21a and pET-28b vectors containing the *cbb*_I_ operon and *cbb*_II_ operon, respectively (11).

**Fig 1:**
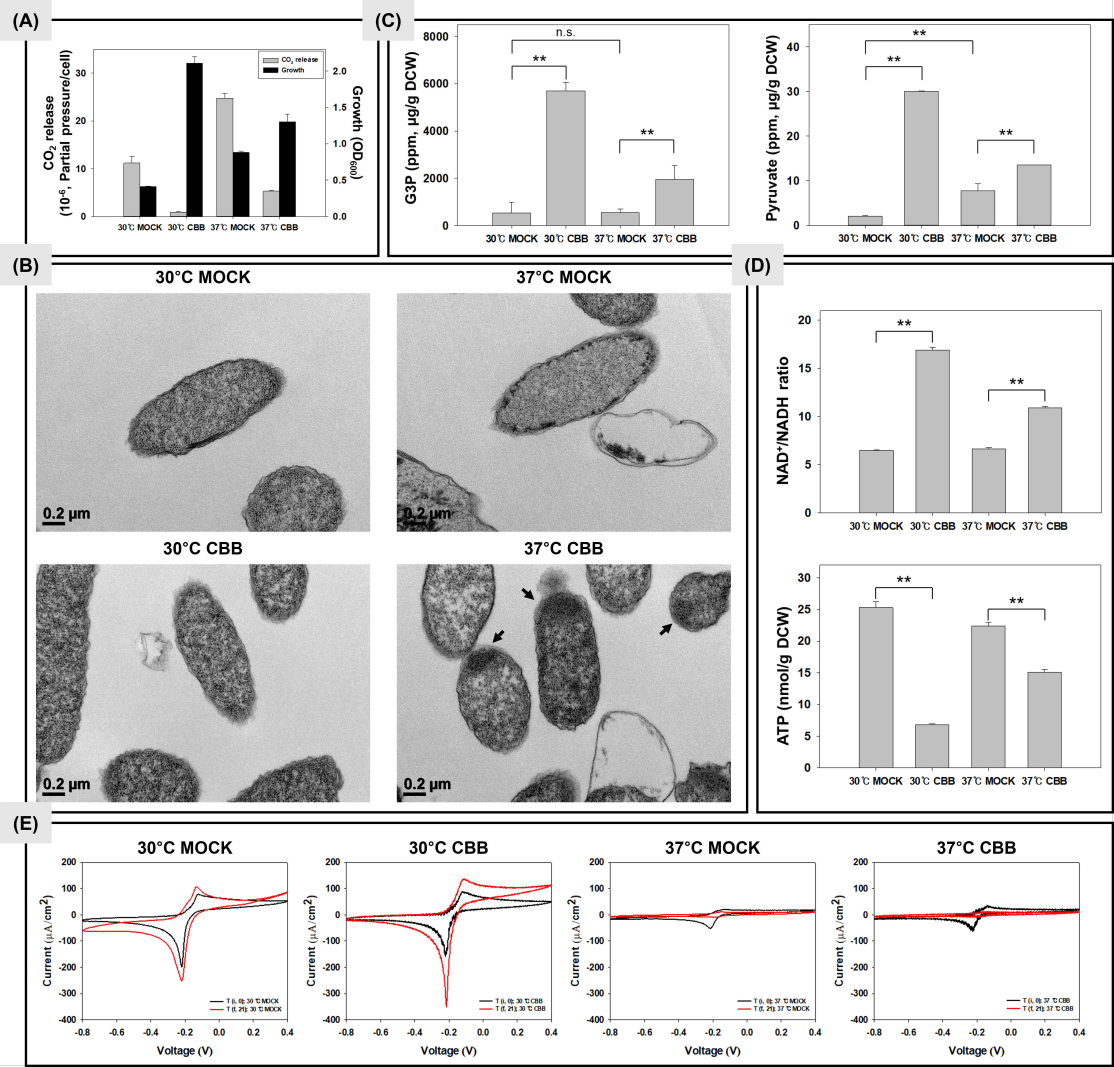
Results evaluated in this study. Control strain (MOCK) and CBB strain containing the CBB genes were used. The results were compared according to the culture temperature. **P < 0.005. (**A**) CO_2_ release amount and cell growth. The CO_2_ values were divided by the number of colony-forming units. (**B**) Transmission electron microscopy images. Black arrows indicate the inclusion bodies. (**C**) The metabolites. The values were divided by dry cell weight (DCW). (**D**) The redox cofactors. ATP concentration was calculated by dividing by DCW. (**E**) Cyclic voltammetry. Scan rate 10 mV s^–1^. T(i) is the initial chronoamperometry point, starting from supplementation with 1 mM isopropyl β-D-1-thiogalactopyranoside and 0.5 mM methylene blue. T(f) is the final chronoamperometry point (*E*_appl_ = –0.2 V vs Ag/AgCl).

Figure. 1A presents the cell growth and measured CO_2_ release from the MOCK and CBB strains at 37°C and 30°C. As a result, at both temperatures, in contrast with the MOCK strain, the CBB strain showed greater bacterial growth, but rather reduced CO_2_ release. Notably, at 30°C, the CBB strain exhibited approximately 5.76 times lower CO_2_ release than at 37°C, highlighting the effectiveness of temperature control in enhancing CO_2_ fixation activity. Next, we confirmed the presence of IB, characterized by dense refractile particles with smooth or irregular rough surfaces, mainly composed of expressed foreign proteins, through the transmission electron microscopy (TEM) images shown in Fig. 1B (12). Consistently, no clear IB was observed in the MOCK strain, whereas clear IB was observed in the CBB strain at 37°C due to heterologous protein expression. However, at 30°C, there was a notable reduction in IB formation in the CBB strain.

Low culture temperature for recombinant protein expression in *E. coli* is a well-established strategy to suppress IB formation (13). At low temperatures, protein solubility is enhanced, and protein aggregation is reduced (14). Therefore, based on these findings, it is evident that temperature control from 37°C to 30°C effectively suppresses IB formation and enhances the endogenous CO_2_ recycling capacity of the CBB strain. On the other hand, at low temperatures, cellular processes slow down, reducing the rate of cell division (15). As a result, it can be confirmed that the growth of the MOCK strain was reduced by low culture temperature. However, the CBB strain showed rather increased growth. It is generally known that the production of recombinant proteins in recombinant strains inhibits cell growth (16), but we mentioned in a previous study that the biomass of the CBB strain was increased through enhanced glycolysis by CO_2_ recycling (11). As such, it can be considered that the biomass of the CBB strain was further increased through increased CO_2_ recycling due to low culture temperature.

### Comparison of the metabolites

Based on the growth results, we analyzed the levels of glyceraldehyde-3-phosphate (G3P) and pyruvate in both the MOCK and CBB strains under different culture temperatures, as shown in Fig. 1C. G3P serves as an intermediate in the CBB pathway and glycolysis (17), while pyruvate is produced through glycolysis from G3P (11). Therefore, when the glycolytic flux is stimulated, the synthesis of G3P and pyruvate can increase. However, lowering the temperature to 30°C did not significantly affect the levels of G3P and pyruvate in the MOCK strain, indicating that temperature reduction did not enhance glycolytic flux.

On the other hand, the CBB strain exhibited higher levels of G3P and pyruvate compared to the MOCK strain at both temperature conditions. Overall, the introduction of the CBB pathway increased the synthesis of organic acids such as pyruvate, which provides reducing power and ATP for carbon fixation (18). This indicates a beneficial cycle of CO_2_ recycling and the synthesis of metabolic intermediates. Importantly, the levels of G3P and pyruvate in the CBB strain were higher at 30°C (5,700.17 ± 349.09 ppm, G3P; 30.04 ± 0.14 ppm, pyruvate) than at 37°C (1,962.20 ± 568.27 ppm, G3P; 13.52 ± 0.01 ppm, pyruvate). These changes in G3P and pyruvate levels were consistent with the CO_2_ release results observed under each condition. Based on these findings, including the growth results of the CBB strain (Fig. 1A), we can predict that temperature control from 37°C to 30°C increased the metabolic rate of the CBB pathway (e.g., G3P synthesis) in the CBB strain, leading to enhanced glycolytic reactions (e.g., pyruvate synthesis).

### Electron consumption during endogenous CO_2_ recycling

Generally, the CBB pathway requires redox cofactors such as NAD(P)H and ATP to facilitate CO_2_ fixation (19, 20). Figure 1D illustrates the changes in ATP concentration and the NAD^+^/NADH ratio in both the MOCK and CBB strains with respect to culture temperature. In comparison to the MOCK strain, the CBB strain exhibited lower levels of ATP and NADH at both temperature conditions. Notably, the reduction in ATP and NADH was more pronounced at 30°C compared to 37°C. Specifically, the ATP concentration in the CBB strain was 6.80 ± 0.14 nmol/g DCW (dry cell weight) at 30°C and 15.09 ± 0.47 nmol/g DCW at 37°C. Furthermore, the NAD^+^/NADH ratio was 16.90 ± 0.28 at 30°C and 10.92 ± 0.12 at 37°C. These changes in ATP and the NAD^+^/NADH ratio correlated with the CO_2_ release results obtained under each temperature condition, indicating that temperature control from 37°C to 30°C enhanced the utilization of electrons from redox cofactors, thereby facilitating increased CO_2_ recycling (11). However, it is important to note that the energy requirements for CO_2_ fixation in the CBB cycle (approximately 9 ATP per carbon atom) exceed the energy provided by glycolysis (approximately 1.7 ATP per carbon atom from G3P to pyruvate). Hence, it should be considered that the CO_2_ fixation in the CBB strain relies on an external energy source, such as glucose.

Next, we compared the electron consumption of the bacteria using bioelectrochemical techniques (Fig. 1E). The bioelectrochemical technique enables the biotransformation and biosynthesis of reduced products by supplying electrons through the cathode to provide reducing power to redox cofactors (21). A single-chamber reactor was used to monitor the current change as a function of voltage. Methylene blue (MB) was utilized to mediate electron transfer to the bacteria, minimizing toxicity and growth inhibition of microorganisms (22). Chronoamperometry and cyclic voltammetry were initiated after isopropyl β-D-1-thiogalactopyranoside (IPTG) induction. In M9 media supplemented with 0.5 mM MB, a peak reduction current was observed around −0.2 V. No significant redox peaks were observed in the MOCK and CBB strains without MB (data not shown). The cyclic voltammetry results demonstrate increased redox currents in the CBB strain compared to the MOCK strain at each temperature. Remarkably, the largest redox current was observed in the CBB strain at 30°C. These significant redox currents indicate improved electrotrophy of the CBB strain, suggesting enhanced electrochemical interactions, such as electron transport between the bacteria and the electrode surface (23). This suggests that more electrons were required for CO_2_ fixation activation and the maintenance of metabolism (24).

### Comparison of protein solubility

**Fig 2:**
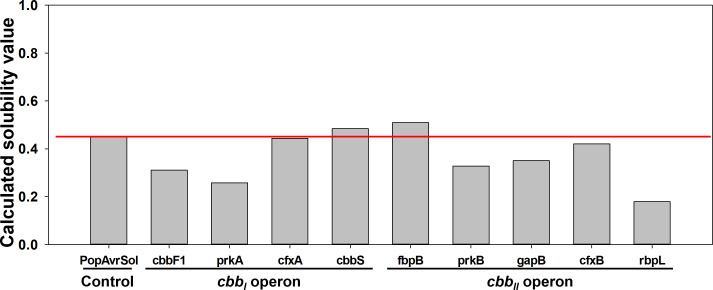
Predicted solubility of proteins expressed from CBB genes. The red line represents the baseline according to the control value of the Protein–Sol web server.

The CBB strain contains the *cbb*_I_ operon (*cbbF1-prkA-cfxA-cbbL-cbbS*) and the *cbb*_II_ operon (*fbpB-prkB-tklB-gapB-cfxB-rbpL*) (25). Both operons contain genes encoding the enzymes fructose-1,6-bisphosphatase (*cbbF1* and *fbpB*) (25), PRK (*prkA* and *prkB*) (26), aldolase (*cfxA* and *cfxB*) (27), and RubisCO (*cbbL*, *cbbS*, and *rbpL*) (28). Additionally, the *cbb*_II_ operon contains genes that encode the enzymes transketolase (*tklB*) (29) and G3P dehydrogenase (*gapB*) (25). The solubility of proteins expressed from CBB genes was predicted using the Protein–Sol web server (http://protein-sol.manchester.ac.uk) to provide clues as to which proteins may be the major contributor to IB formation (Fig. 2). Protein–Sol is a web server that predicts protein solubility through the calculation of 35 sequence-based properties using data for protein solubility of *E. coli* in a cell-free expression system (30). Predicted solubility is expressed as a value from 0 to 1, and an experimental data set of the average soluble *E. coli* protein represented by PopAvrSol is used as a control (31). Thus, a value higher than the control value (0.45) means predicted to have a higher solubility than the average soluble *E. coli* protein; conversely, a lower value means predicted to be less soluble (30). As a result, out of a total of 11 proteins, two proteins were predicted to have higher solubility (0.484, cbbS; 0.509, fbpB) and seven proteins to have lower solubility (0.311, cbbF1; 0.258, prkA; 0.444, cfxA; 0.328, prkB; 0.350, gapB; 0.420, cfxB; 0.179, rbpL) than the average soluble *E. coli* protein. The other two proteins’ solubility (cbbL and tklB) could not be predicted because they were predicted to have a transmembrane segment (30). Comprehensively, based on protein solubility prediction results, cfxB is expected to have the most significant effect on IB formation, followed by prkA, cbbF1, cfxA, prkB, gapB, and prkA.

### Conclusion

We conducted this study to investigate the impact of culture temperature on CO_2_ recycling in the CBB strain. By reducing the culture temperature from 37°C to 30°C, we successfully suppressed IB formation, leading to a significant reduction in CO_2_ release by approximately 5.76 times. Moreover, we observed a simultaneous increase in pyruvate accumulation by approximately 2.3 times. These results demonstrate the effectiveness of temperature control in improving CO_2_ recycling and organic acid synthesis. Based on these findings, we believe that temperature control holds promise as an approach to enhance chemical biosynthetic productivity and biological CO_2_ fixation in integrated autotrophic biorefinery strategies. However, it is important to note that maximizing the carbon fixation capacity of the CBB strain is a complex challenge. In this study, we focused on temperature control, but it is worth considering that other environmental factors, such as IPTG concentration and pH, can influence IB formation in the recombinant strain. Additionally, the interplay of multiple environmental conditions can impact the overall system. Future research will explore the optimization of various environmental parameters to maximize carbon fixation activity. In conclusion, temperature control represents a valuable avenue for improving CO_2_ recycling and organic acid synthesis in the CBB strain, offering potential benefits for advancing integrated autotrophic biorefinery strategies.

## MATERIALS AND METHODS

### Bacterial strain and growth conditions

The bacteria were grown in 40 mL of M9 minimal medium (3 g L^–1^ KH_2_PO_4_, 1 g L^–1^ NH_4_Cl, 12.8 g L^–1^ Na_2_HPO_4_·7H_2_O, 0.1 mM CaCl_2_, 2 mM MgSO_4_, 0.5 g L^−1^ NaOH, and 4 g L^−1^ glucose) with 50 ng L^–1^ ampicillin and 50 ng L^–1^ kanamycin. The bacteria were cultured in a 100 mL serum bottle at 180 rpm and each temperature under aerobic conditions. After 3 h of incubation (optical density at 600 nm [OD_600_], ~0.4), 1 mM IPTG was added to induce heterogeneous expression. After IPTG induction, the cultures were tightly capped with a gas-tight rubber stopper for semi-anaerobic cultures. Unless otherwise noted, both strains were incubated for 24 h. The growth of the cells was monitored by measuring the optical density at 600 nm with sufficient dilution using an ultraviolet-visible (UV-Vis) spectrophotometer (Mecasys Co., LTD., Daejeon, Korea).

### Gas chromatography (GC)

The amount of CO_2_ release was estimated by YL6500 GC (YOUNGIN Chromass Co., Ltd., Korea) equipped with a 60/80 carboxen-1000 column (Supelco, Bellefonte, PA, USA) and a thermal conductivity detector. The oven was set at 150°C, and the temperature of the injector and detector was 70°C and 180°C, respectively. N_2_ was used as the carrier gas. The CO_2_ release was calculated as the amount of CO_2_ release divided by the number of the colony-forming unit.

### Transmission electron microscopy

The methods from primary fixation to ethanol dehydration were performed according to a previously published protocol (32). The dehydrated samples were transitioned twice with 100% propylene oxide for 5 min at room temperature. For infiltration, the samples were added to a resin series (resin:propylene oxide = 1:1 for 2 h; resin:propylene oxide = 3:1 overnight; 100% resin for 2 h). The samples were centrifuged at 10,000 rpm for 1 min in each step. After infiltration, the samples were transferred to a mold and polymerized at 60°C for 48 h by adding 100% resin. The samples were cut into ultra-thin sections of ~70 nm using an ultramicrotome (Leica EM UC7/FC7; Leica Microsystems, Wetzlar, Germany). Next, the samples were double-strained with 2% uranyl acetate and 2% Reynold’s lead citrate for 7 min each on a 200-mesh grid. The samples were washed with distilled water seven times for 1 min each between each staining. The samples were observed by TEM (Hitachi H-7650; Hitachi High-Technologies, Canada) under an accelerating voltage of 100 kV.

### Liquid chromatography with tandem mass spectrometry (LC/MS/MS)

According to a previously published protocol, LC/MS/MS analysis was performed (32). Pyruvate standard was purchased from Sigma-Aldrich (St. Louis, MO, USA). G3P standard was purchased from the Cayman Chemical Company (Ann Arbor, MI, USA). Standard solutions of pyruvate and G3P were prepared using analytical-grade ethanol.

### NAD^+^/NADH and ATP assay

NADH and NAD^+^ were detected using a NAD/NADH-Glo Assay kit (Promega Co., Ltd, Fitchburg, WI, USA). NADH and NAD^+^ were extracted using formulated extraction solutions (0.4 N HCl, 0.2 N NaOH/20% DTAB, 0.2 N HCl/0.25 M Trizma, and 0.5 M Trizma). The luminescence of a mixture of 100 μL of the NAD/NADH detection reagents and 100 μL of the sample was detected using the GloMax Explorer system (Promega Co.). ATP was detected using a BacTiter-Glo microbial cell viability assay kit (Promega Co.). An ATP standard curve was generated from 10^–12^ to 10^–9^ M. The luminescence of a mixture of 100 μL of reagents and 100 μL of the sample was detected using a GloMax Explorer system.

### Bioelectrochemical analysis

The method of bioelectrochemical analysis was based on previously published protocols with some modifications (11). Electrochemical measurements were performed after purging with Ar for 15 min to remove oxygen from the electrolyte (M9 minimal medium).

### Prediction of protein solubility

The solubility of proteins was predicted using the Protein–Sol web server. Amino acid sequences for solubility prediction were obtained through National Center for Biotechnology Information (NCBI)’s database (RSP_1285, cbbF1; RSP_1284, prkA; RSP_1283, cfxA; RSP_1282, cbbL; RSP_1281, cbbS; RSP_3266, fbpB; RSP_3267, prkB; RSP_3268, tklB; RSP_3269, gapB; RSP_3270, cfxB; RSP_3271, rbpL).

### Statistical analysis

All experiments were performed independently in triplicate for error analysis. The results are shown along with the correlation and standard deviation for the experimental conditions. The experimental data were analyzed using Sigma Plot (Systat Software Inc., San Jose, CA, USA).

## Data Availability

All data generated or analyzed in this study are included in this article.
